# Therapeutic applications of synthetic gene/genetic circuits: a patent review

**DOI:** 10.3389/fbioe.2024.1425529

**Published:** 2024-08-05

**Authors:** Diego C. Carneiro, Vinícius P. C. Rocha, Patrícia K. F. Damasceno, Josiane D. V. Barbosa, Milena B. P. Soares

**Affiliations:** ^1^ Gonçalo Moniz Institute, Oswaldo Cruz Foundation, Salvador, Brazil; ^2^ SENAI Institute of Advanced Health Systems Innovation, University Center SENAI CIMATEC, Salvador, Brazil

**Keywords:** synthetic biology, genetic engineering, gene networks, intellectual property, molecular circuits, biosensors, genetic switch

## Abstract

A significant limitation of numerous current genetic engineering therapy approaches is their limited control over the strength, timing, or cellular context of their therapeutic effect. Synthetic gene/genetic circuits are synthetic biology approaches that can control the generation, transformation, or depletion of a specific DNA, RNA, or protein and provide precise control over gene expression and cellular behavior. They can be designed to perform logical operations by carefully selecting promoters, repressors, and other genetic components. Patent search was performed in Espacenet, resulting in 38 selected patents with 15 most frequent international classifications. Patent embodiments were categorized into applications for the delivery of therapeutic molecules, treatment of infectious diseases, treatment of cancer, treatment of bleeding, and treatment of metabolic disorders. The logic gates of selected genetic circuits are described to comprehensively demonstrate their therapeutic applications. Synthetic gene circuits can be customized for precise control of therapeutic interventions, leading to personalized therapies that respond specifically to individual patient needs, enhancing treatment efficacy and minimizing side effects. They can be highly sensitive biosensors that provide real-time therapy by accurate monitoring various biomarkers or pathogens and appropriately synthesizing a therapeutic molecule. Synthetic gene circuits may also lead to the development of advanced regenerative therapies and to implantable biodevices that produce on-demand bioactive molecules. However, this technology faces challenges for commercial profitability. The genetic circuit designs need adjustments for specific applications, and may have disadvantages like toxicity from multiple regulators, homologous recombination, context dependency, resource overuse, and environmental variability.

## Introduction

Cells respond to particular signals by initiating gene expression, enabling them to navigate microenvironments, communicate with other cells, and form intricate patterns. Genetic engineers aim to leverage this ability by programming cells to undertake tasks or produce useful chemicals and materials. Synthetic gene/genetic circuits are constructed through the adaptation of a set of regulators and genetic components as modular parts. They empower engineers to create intricate systems by integrating distinct functional units with well-defined inputs and outputs (Brophy and Voigt, 2014). A significant limitation of numerous current therapeutic approaches in genetic engineering is their limited control over the strength, timing, or cellular context of the therapeutic effect. Synthetic gene circuits have the capacity to control the generation, transformation, or depletion of specific DNA, RNA, or protein molecules. Gene and engineered-cell therapies can enhance a cell’s genetic program, aiming to alter its behavior for therapeutic, preventive and diagnostic purposes. The overall behavior of a genetic circuit is articulated as a comprehensive array of logic operations, and they can be broken down into modules containing relevant logical relationships (Kitada et al., 2018).

Inspired by synthetic biology, cell-based therapies incorporating therapeutic gene circuits for on-demand restoration of homeostasis may revolutionize medicine. The disease-control attributes of synthetic genetic circuits encompass preventing diseases, and identifying diseases with/without subsequent initiation of an autonomously regulated therapeutic response (Sedlmayer et al., 2018). Gene switches empower mammalian regulators to finely modulate protein synthesis across various gene expression levels. This includes transcription initiation, gene editing, RNA splicing regulation, protein translation regulation, interference RNA (RNAi) and antisense technology for gene knockdown, and the management of protein localization and interactions. Both inputs and outputs of genetic circuits are usually proteins or nucleic acids, but small ligands, light or heat can also serve as triggers to induce regulatory molecular interactions (Xie and Fussenegger, 2018). In this review, we aim to navigate through the state-of-the-art of the therapeutic applications of synthetic gene circuit present in the literature of patents, highlighting the prospects of this emerging technology to urge its development in the medical industry.

### Patent search

Patents were initially retrieved from Espacenet, the European Patent Office search service (https://worldwide.espacenet.com/patent/) in June 2024, using the advanced tool with the following search keywords and Boolean operators: ta = “gene circuit” OR ta = “genetic circuit” OR ta = “gene circuits” OR ta = “genetic circuits,” to find patents using those terms to designate this technology. The search was limited to the title or abstract fields and not limited to a timeframe. This strategy found 145 results from which the information in the bibliographic data and summaries was verified so that duplications and documents without description of therapeutic applications of synthetic gene circuits were discarded, resulting in 29 patents included in this review, as listed in Table 1.

**TABLE 1:** List of the main findings from the patents initially retrieved from Espacenet.

N°	Title	Applicants	Publication number	Publication date	IPC
1	Engineered phagemids	Massachusetts Inst Technology [US] Univ Boston [US] Harvard College [US]	US11473104B2 US2018355378A1	2018-12-13 2022-10-18	A61K35/76 C07K14/245 C07K14/435 C07K14/46 C12N15/70 C12N15/86 C07K14/35C12N15/63 C12N15/74 C07K14/61
2	Gene circuit, RNA delivery system and use thereof	Univ Nanjing [CN]	WO2022206734A1	2022-10-06	A61K31/713 A61P25/30 C12N15/113 C12N15/63
3	Programming living glue systems to perform autonomous mechanical repairs	Univ Shanghai Tech [CN]	US2023220370A1	2023-07-13	C07K14/245 C07K14/435 C09J189/00 C12N11/04 C12N15/63 C12N15/70 C12N9/02
4	Genetic circuit inverting amplifier	Davies Stephen W Derubertis Gianna [US]	US2005112615A1	2005-05-26	C12Q1/68 H03F21/00 H03F99/00
5	Self-surface-releasing bacterial treatment method based on synthetic circuits and protein engineering	Bilkent Univ Ulusal Nanoteknoloji Arastirma Merkezi [TR]	WO2023128983A1	2023-07-06	A61K35/741 C12N15/74 C12N5/10 C12N9/10
6	Photonic nanoantenna mediated gene circuit reconfiguration	Univ California [US]	US2014342427A1	2014-11-20	C12N13/00
7	Compositions and methods for the *in situ* delivery of therapeutic and diagnostic agents	Synlife Inc [US]	US2022049269A1	2022-02-17	C12N15/85
8	Regulated expression of cloned genes using a cascade genetic circuit	Consejo Superior de Investigaciones Científicas [ES]	US2001016354A1 US6803224B2	2001-08-23 2004-10-12	C12N15/63 C07H21/04 C12N1/21 C12N1/21 C12N15/67 C12N15/70
9	High performance multi-input microrna sensors and uses thereof	Massachusetts Inst Technology [US]	US11359247B2 US2019032141A1 WO2019027414A1 WO2019027414A8	2019-01-31 2022-06-14	C12Q1/68 A61K31/7125 C12N15/113 C12Q1/6811 C12Q1/6886 C12Q1/6897
10	Transgene expression system	Univ Court Univ of Edinburgh [GB]	US2023323391A1	2023-10-12	C12N15/11 C12N15/86
11	Thermal control of T cell immunotherapy by molecular and physical initiation	California Inst of Techn [CN]	CN115768890A	2023-03-07	A61K35/17 A61N2/00 A61N5/02 A61N5/06 A61N7/02 A61P35/00 C12N15/10 C12N15/63 C12N5/0783
12	Genetic indicator and control system and method utilizing split Cas9/CRISPR domains for transcriptional control in eukaryotic cell lines	Univ Tsinghua [CN]	US2017233703A1	2017-08-17	A61K48/00 C12N15/82 C12N15/86 C12N7/00 C12N9/22
13	Methods and compositions for RNA-guided genetic circuits	Massachusetts Inst Technology [US]	US2019345501A1	2019-11-14	C12N15/11 C12N15/63 C12N15/70 C12N9/22
14	Engineered dCas9 with reduced toxicity and its use in genetic circuits	Massachusetts Inst Technology [US]	US2020095589A1 US2021079404A9	2020-03-26 2021-03-18	C07K14/37 C12N15/62 C12N9/02 C12N9/22
15	RNA-based logic circuits with RNA binding proteins, aptamers and small molecules	Massachusetts Inst Technology [US] Univ Kyoto [JP] Weiss Ron [US] Wroblewska Liliana [US] Siciliano Velia [US] Kitada Tasuku [BE] Hottelet Foley Maria [US] Bodner Katie [US] Saito Hirohide [JP] Endo Kei [JP] Irvine Darrell J [US] Wagner Tyler [US] Becraft Jacob [US]	US11351271B2 US2018296702A1 US2019151474A2	2018-10-18 2019-05-23 2022-06-07	A61K48/00 C12N15/85 C12N15/10 C12N15/11 C12N15/63
16	Engineered hypoxia biosensors and methods of using the same	Univ Northwestern [US]	WO2024015383A1	2024-01-18	C12N15/85
17	Acoustic remote control of microbial immunotherapy	California Inst Of Techn [US]	WO2022212005A2 WO2022212005A3 WO2022212005A9	2022-10-06 2022-12-22 2022-11-17	C12N15/74 A61K35/74 C12N13/00 C12N15/70
18	Genetically engineered sensors for *in vivo* detection of bleeding	Massachusetts Inst Technology [US]	US2017058282A1	2017-03-02	A61K49/00 C12N15/63 C12N15/70 C12Q1/02 C12Q1/68
19	Thermal state switches in macrophages	California Inst Of Techn [US]	US2023103980A1	2023-04-06	B01L7/00 C12N15/63 C12N15/82 G01N33/53
20	Methods of engineering platelets for targeting circulating tumor cells	Univ Utah Res Found [US]	US2022348938A1	2022-11-03	A61K35/19 A61P35/00 C07K16/30 C12N15/63 C12N5/078
21	T cell activation responsive constructs for enhanced CAR-T cell therapy	Univ California [US]	WO2023225482A2 WO2023225482A3	2023-11-23 2023-12-21	A61K38/16 C12N15/85 A61K35/17 C07K16/18 A61P35/00 C07K16/30
22	Tumor immunotherapy	Massachusetts Inst Technology [US]	WO2016205737A2 WO2016205737A3	2016-12-22 2017-02-02	G01N33/68 C12Q1/68 A61K39/395 C12N15/00 C12N15/10 C12N15/11 C12N15/113 C12N15/63
23	Systems and methods for control of gene expression	Univ Boston [US]	US11781149B2 US2020002710A1	2020-01-02 2023-10-10	C07K14/47 C12N15/82
24	Autocatalytic base editing for RNA-responsive translational control	Massachusetts Inst Technology [US] Harvard College [US]	US2024067957A1	2024-02-29	C12N15/10 C12N15/11 C12N15/86 C12N9/78
25	Red light-regulated transcriptional activation device/system for mammals, and construction method therefor and use thereof in gene therapy	Univ East China Normal [CN]	WO2023231931A1	2023-12-07	A61K38/28 A61P35/00 C12N15/864
26	Topologies of synthetic gene circuit for optimal fold change activation	Technion Res and Dev Foundation [IL]	US2023130375A1	2023-04-27	A61K48/00 C12N15/63
27	Synthetic hybrid receptor and genetic circuit in bacteria to detect enteric pathogenic microorganisms	Massachusetts Inst Technology [US] Univ Boston [US]	US10802021B2 US2018328923A1 US2020173993A9	2018-11-15 2020-06-04 2020-10-13	C07K14/28 G01N33/569 A61K35/744 C07K14/195 C12N15/62 C12N9/12
28	Chimeric antigen receptor cell library carrying gene element combination, preparation and screening method, and use thereof	Pharchoice Therapeutics Inc [CN]	WO2021093484A1	2021-05-20	A61K39/00 A61P29/00 A61P35/00 C12N15/85 C12N5/10 C40B40/02 C40B50/06
29	Compositions and methods for electronic control of gene expression	Univ Maryland [US]	WO2018106932A2 WO2018106932A3	2018-06-14 2018-07-12	C12Q1/00 C12Q1/6825 C12N15/70 G01N33/50

Then, Google Scholar (https://scholar.google.com/) was used to search articles with the following search terms and Boolean operators: (gene circuit OR genetic circuit OR molecular circuit OR switch OR biosensor OR sensor) AND (therapeutic OR therapy OR treatment). The most relevant original articles were selected, and the names from the first and last authors were used as keywords with the AND operator in the inventors search field from Espacenet to retrieve the articles’ derived patents. Duplications were discarded, resulting in 9 patents, as listed in Table 2.

**TABLE 2:** List of the main findings from the Google Scholar and Espacenet search.

N°	Title	Applicants	Publication number	Publication date	IPC	Article reference
1	Designer circuit controlling diet-induced obesity	ETH Zuerich [CH]	WO2014117945A2 WO2014117945A3	2014-08-07 2014-10-23	C12N5/00	Rössger et al. (2013)
2	Composition for treatment of tuberculosis	ETH Zuerich [CH] Fussenegger Martin [CH] Weber Wilfried [CH] Schoenmakers Ronald [NL]	WO2009080432A2 WO2009080432A3	2009-07-02 2010-02-18	A61K31/4409 A61K45/06 A61P31/06	Weber et al. (2008)
3	Control of uric acid homeostasis	ETH Zuerich [CH] Kemmer Christian [CH] Weber Wilfried [DE] Fussenegger Martin [CH]	WO2010133298A1	2010-11-25	A61K38/44 C12N15/63 C12N9/06 C12Q1/68	Kemme et al. (2010)
4	Programmable oncolytic virus vaccine system and application thereof	Univ Tsinghua [CN] Beijing Syngentech Co Ltd [CN]	WO2018171103A1	2018-09-27	A61K35/00 A61K35/761 A61K47/00 A61K47/46 A61K48/00 A61P35/00 C12N15/00 C12N15/11 C12N15/113 C12N15/861 C12N7/00 C12N7/01	Huang et al. (2019)
5	Compositions and methods for cell-based delivery system	Univ Washington [US] Shriners Hospitals Children [US]	WO2022212754A1	2022-10-06	C07K14/47 C07K14/475 C12N15/12 C12N5/10	Pferdehirt et al. (2019)
6	Methods for generating mechanically-responsive cells and uses thereof	Univ Washington [US] Washington University St Louis [US] Shriners Hospitals Children [US]	WO2022198126A1 WO2022198126A8 WO2022198126A9	2022-09-22 2023-11-09 2022-10-27	A61K48/00 A61L27/58	Nims et al. (2021)
7	Endogenous gene regulation to treat neurological disorders and diseases	UCL Business Ltd [GB] Ospedale San Raffaela S R L [IT]	WO2023131682A1	2023-07-13	C12N15/113	Qiu et al. (2022)
8	Recombinase-based logic and memory systems	Massachusetts Inst Technology [US]	WO2014093852A1	2014-06-19	C12N15/09 C12N15/63 G06N3/12	Siuti et al. (2013)
9	Gene system for identifying P53 mutation specifically	2nd Peoples Hospital Shenzhen [CN]	CN108728441A CN108728441B	2018-11-02 2022-07-22	C12N15/113 C12Q1/6886	Zhan et al. (2018)

### Classification and geographical distribution

The international patent classification (IPC) codes provided by the patent documents were used to retrieve their fields and descriptions from the World Intellectual Property Organization (WIPO) service (https://www.wipo.int/classifications/ipc/en/), resulting in 29 categories, according to Table 3. The IPC C12N15 was present in 34 out of 38 patents selected. Most patent applicants were from United States (57.9%), China (21%), and Switzerland (8%), followed by United Kingdom (5.3%), Israel (2.6%), Spain (2.6%), and Turkey (2.6%).

**TABLE 3:** Most frequent international patent classification of the patents included in this review.

Field	IPC	Description	Number of patents
PREPARATIONS FOR MEDICAL, DENTAL OR TOILETRY PURPOSES	A61K35	Medicinal preparations containing materials or reaction products thereof with undetermined constitution	8
	A61K31	Medicinal preparations containing organic active ingredients	3
	A61K38	Medicinal preparations containing peptides	3
	A61K39	Medicinal preparations containing antigens or antibodies	2
	A61K48	Medicinal preparations containing genetic material which is inserted into cells of the living body to treat genetic diseases; Gene therapy	5
SPECIFIC THERAPEUTIC ACTIVITY OF CHEMICAL COMPOUNDS OR MEDICINAL PREPARATIONS	A61P35	Antineoplastic agents	6
PEPTIDES	C07K14	Peptides having more than 20 amino acids; Gastrins; Somatostatins; Melanotropins; Derivatives thereof	7
	C07K16	Immunoglobulins	2
	C12N13	Treatment of microorganisms or enzymes with electrical or wave energy, sonic waves	2
	C12N15	Mutation or genetic engineering; DNA or RNA concerning genetic engineering, vectors, or their isolation, preparation or purification; Use of hosts therefor	34
	C12N5	Undifferentiated human, animal or plant cells; Tissues; Cultivation or maintenance thereof; Culture media therefor	6
	C12N7	Viruses; Compositions thereof; Preparation or purification thereof; preparing medicinal viral antigen or antibody compositions	2
	C12N9	Enzymes; Proenzymes; Compositions thereof	8
MEASURING OR TESTING PROCESSES INVOLVING ENZYMES, NUCLEIC ACIDS OR MICROORGANISMS; COMPOSITIONS OR TEST PAPERS THEREFOR; PROCESSES OF PREPARING SUCH COMPOSITIONS; CONDITION-RESPONSIVE CONTROL IN MICROBIOLOGICAL OR ENZYMOLOGICAL PROCESSES	C12Q1	Measuring or testing processes involving enzymes, nucleic acids or microorganisms; Compositions therefor; Processes of preparing such compositions	7
INVESTIGATING OR ANALYSING MATERIALS BY DETERMINING THEIR CHEMICAL OR PHYSICAL PROPERTIES	G01N33	Investigating or analysing materials by specific methods not covered by groups	5

### Therapeutic applications

**FIGURE 1:**
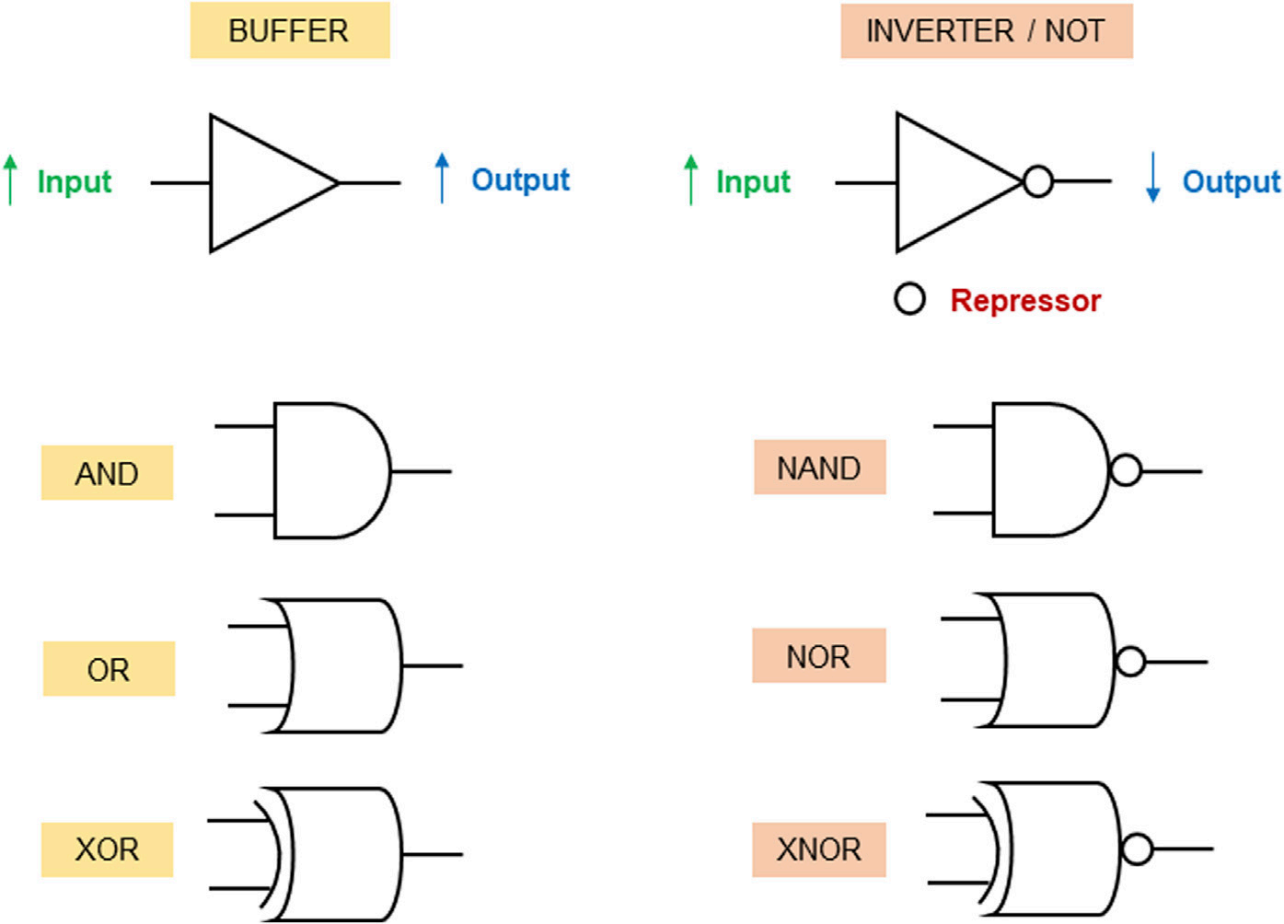
Genetic circuit logic gates. While increase of input signals in BUFFER gates induces output expression, a repressor is used to inhibit the expression of the output gene when the input levels increase in NOT gates (inverters). AND gate: the output gene is activated only when all inputs are present. OR gate: the output gene is activated when at least one of the inputs are present. XOR gate: the output gene is activated when exactly one of the inputs is present. NAND gate: the output is expressed only when all inputs are not present. NOR gate: the output gene is expressed only when at least one of the inputs are not present. XNOR gate: the output is expressed only when exactly all inputs are either present or absent.

Synthetic gene/genetic circuit logic gates operate by utilizing principles of synthetic biology to design biological systems that can process and transmit genetic information in a manner analogous to electronic logic gates in traditional computers. They are constructed by designing the regulatory interactions between genes and the elements that control gene expression. By carefully selecting promoters, repressors, and other genetic components, genetic circuits can be created to perform logical operations at the molecular level (Goñi-Moreno et al., 2011). The synthetic gene circuit’s structure involves a sensor (input) as the first layer, a “processor” managing signals, and an output layer with regulated genes, influencing cell functionalities (Xia et al., 2019). Figure 1 describes the symbols and functions of genetic circuit logic gates. In this review, we use the logic gates symbols to describe the therapeutic applications of synthetic gene circuits in the patent literature. We grouped the applications as embodiments for the delivery of therapeutic molecules (proteins, peptides or RNAs), treatment of infectious diseases, treatment of cancer, treatment of bleeding, and treatment of metabolic disorders. Those embodiments are described in the following sections.

### Delivery of therapeutic molecules

**FIGURE 2:**
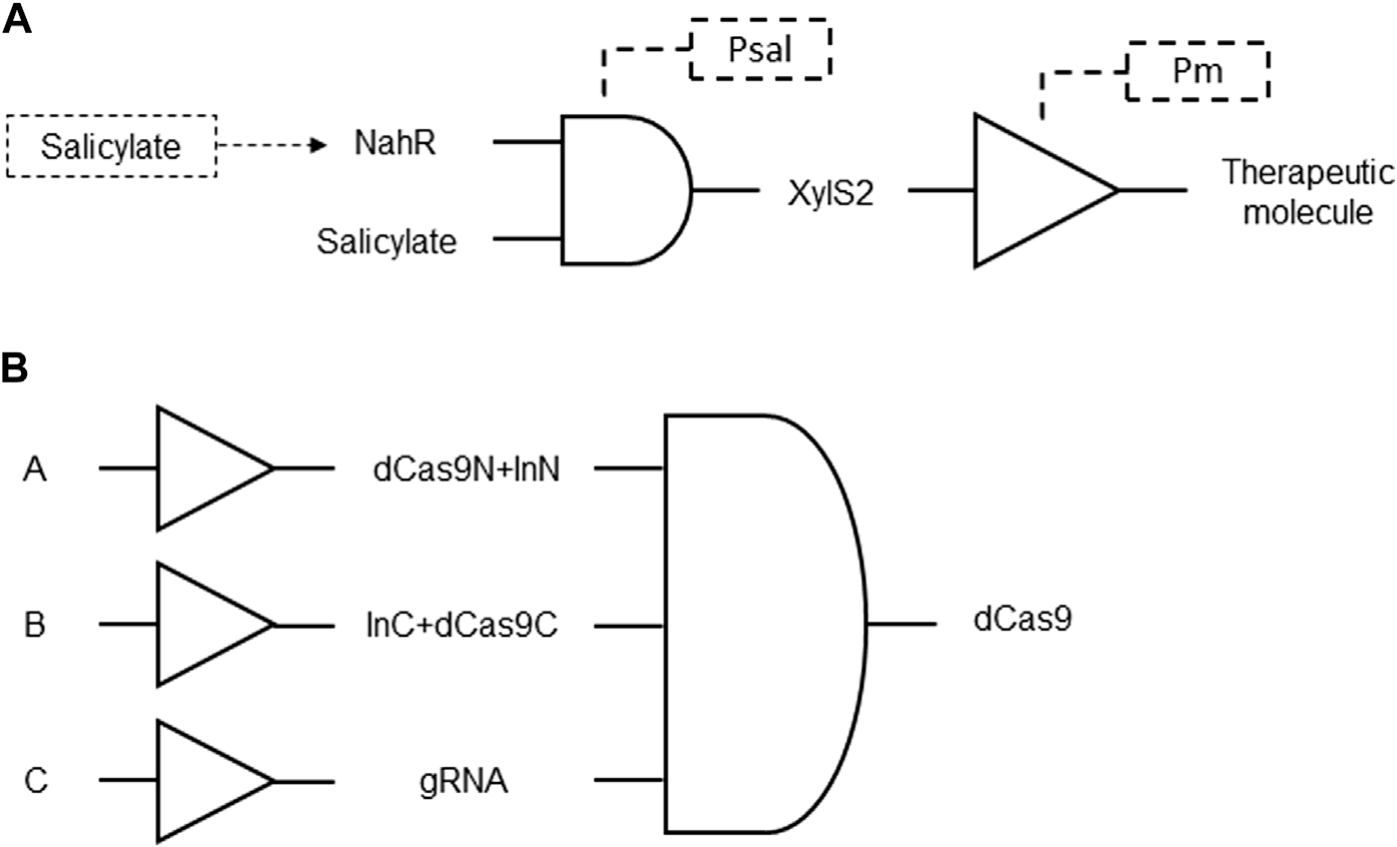
**(A)** Delivery of therapeutic proteins, peptides or RNAs by a cascade genetic circuit using AND gate coupled NahR and salicylate expression of Pm inducer XylS2. The expression of XylS2 is under the control of the Psal promoter that is activated by salicylate. NahR transcriptional activator also regulates the expression of xylS2 and is also activated in response to salicylate. XylS2 is a regulatory protein that influences the activation of the Pm promoter. With this coupled elevated concentration of XylS2 protein, the activation of the Pm promoter is strengthened and, consequently, also the expression of therapeutic target genes. **(B)** dCas9 delivery by a split AND gate gene circuit. Sensory switches with generic inputs A, B, and C induce expression of intein-mediated (InN and InC) dCas9 domains (dCas9N and dCas9C) and guide RNA (gRNA) that are reconstituted to form full-length dCas9.

A genetic circuit was developed with a cascade system designed to achieve significantly higher gene expression, up to 10 to 20 times more than the standard nahR/Psal system, while maintaining the advantages of the latter. This cascade circuit incorporates the regulatory element xylS2 and its target promoter Pm in conjunction with Psal expression. The xylS2 gene responds to a common inducer, such as salicylate, and exhibits greater gene expression capacity than the standard nahR/Psal system (Figure 2A). The cascade system works synergistically, as the XylS2 transcriptional activator enhances the activation of the Pm promoter. It includes hierarchical transcriptional regulators and a final target promoter responsive to a terminal downstream regulator. Methods for regulating nucleic acid sequence expression using this cascade genetic circuit are provided, with applications in various cell types and organisms (Angel et al., 2001; Table 1 patent 8).

To overcome limitations in the delivery and size of CRISPR/Cas systems, particularly in mammalian cells, an innovative approach was developed using a genetic circuit. Traditional strategies for engineering gene circuits in mammalian cells using dCas9 and gRNA expression face challenges, such as restrictive cargo size in viral delivery vehicles. The invention introduces a split Cas9 system, where dCas9 is split and reconstituted in human cells, enabling the retention of powerful genetic manipulation functionality with increased space for cellular programming purposes. This split Cas9 system is integrated into a cascade genetic circuit, allowing for modular and efficient construction of complex logic AND circuits (Figure 2B). By employing intein-mediated split Cas9, the coding sequence of Cas9 is distributed across multiple vectors and reconstituted post-translationally. The system provides higher-level control by fusing Cas9 fragments with regulatory domains and demonstrates successful applications in bladder cancer cells, showcasing specificity for diagnostic and potential therapeutic purposes. The invention expands the capabilities of synthetic biology and CRISPR/Cas systems, offering improvements in genetic circuitry, delivery systems, and regulatory control for biomedical applications (Zhen et al., 2017; Table 1 patent 12).

**FIGURE 3:**
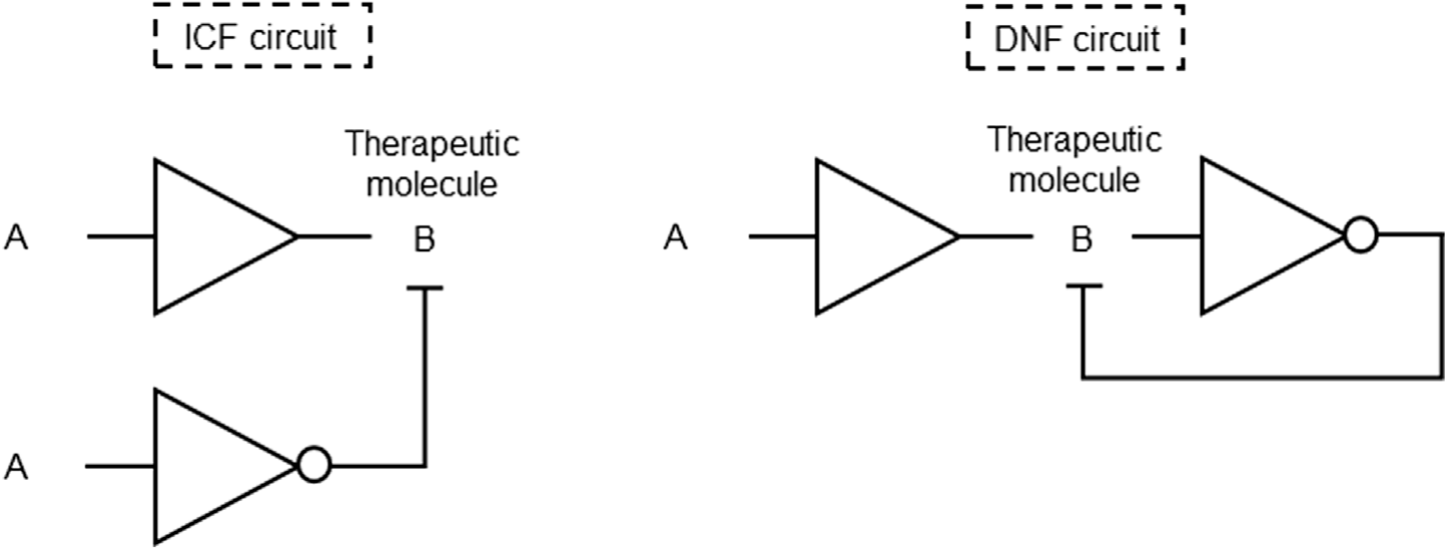
Gene circuits formed by BUFFER and NOT gates for fine-tuned control of therapeutic genes. Generic input A regulate both the output B (therapeutic molecule) and its inhibitor in the ICF circuit. Generic input A induces repression of its output B by an inhibitor of B in the DNF circuit.

Genetic circuit versatile systems for controlling gene expression in cells using expression vectors and feedback mechanisms was designed. This invention enables fine-tuned control over gene expression of therapeutic molecules and provides an effective tool for reducing basal expression levels while maintaining high expression in the presence of a desired input signal. The fold change activation is characterized by the transfer function of promoter activity resulted from the ratio between ON and OFF states. The described system involves two distinct designs for controlling genetic switches: the indirect coherent feedforward (ICF) and double negative feedback (DNF) systems. In the ICF circuit, input molecules regulate both the inhibitor and the output level. This circuit involves a positive regulation between the two branches, where the difference determines the circuit output. On the other hand, the DNF circuit employs mutual inhibition through a negative feedback loop formed by an inverter with a specific gain (Figure 3). Various embodiments and applications are described, showcasing the versatility of the system in regulating gene expression (Ramez et al., 2023; Table 1 patent 16).

Another invention proposed a genetic circuit amplifier analogous to an inverting amplifier in electronics. It produces an output where an increase in the input signal results in a proportional decrease in the output and *vice versa*. The key innovation in this invention is the application of negative feedback to counter the influence of the input signal, ensuring that the change in output is more proportionate to the change in input. Amplifiers with negative feedback in genetic circuits offer advantages such as improved signal-to-noise ratio, stability, and accuracy. This technology may find applications in investigating cell differentiation and cancer within intracellular environments (Davies Stephen and Gianna, 2005; Table 1 patent 4).

**FIGURE 4:**
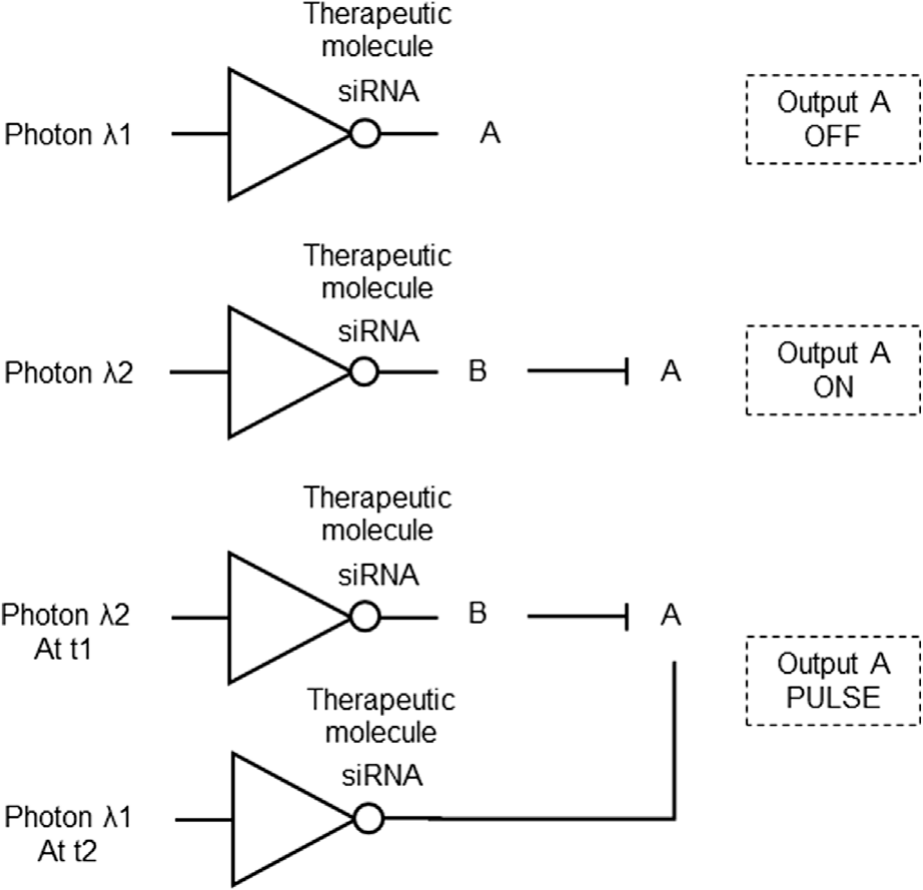
Optical gene circuit system constructed with NOT logic gates. Top: A biomolecular nanoantenna optically addressed by photon λ1 emits a siRNA (therapeutic molecule) as the repressor that turns OFF output A expression. Middle: A biomolecular nanoantenna optically addressed by photon λ2 emits a siRNA that silences output B, which is an inhibitor of A, turning ON output A. Bottom: The biomolecular nanoantennas optically addressed by photons λ1 and λ2 are combined, turning ON output c expression at time t1 when photon λ2 is the input, and turning OFF output A expression at time t2 when photon λ1 is the input, producing an output A PULSE.

To address the need for precise and controllable biomolecular carriers and provide a method for on/off-switch control and engineering of photonic gene circuits, an on-demand optical gene circuit system was proposed. It uses resonant optical nanoantennas that act as selectively addressable optical receivers and biomolecular emitters within cells. Rod-shaped gold nanoantennas efficiently convert absorbed optical energy into surface-localized heat, releasing biomolecules functionalized to the nanoantenna’s surface. The surface of the nanoantenna is modified with a cationic lipid layer, facilitating its introduction into cells and adsorption of biomolecules, such as negatively charged siRNA, forming a biomolecular nanoantenna carrier. The nanoantenna functions as an optical receiver and biomolecular emitter, releasing siRNA or other biomolecules when the antenna effect is “on” at the resonance state. This technology enables dynamic optical circuit reconfiguration, forming photonic gene circuits with precise control over gene modulation (Figure 4). It has potential applications in probing, identifying, and reconfiguring malfunctioning gene expression implicated in disease progression and cancer, by providing photonic and temporal control over biomolecule release for gene modulation (Lee and Lee, 2014; Table 1 patent 6).

**FIGURE 5:**
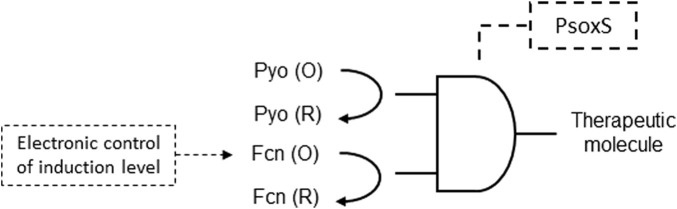
Electrogenetic gene circuit device constructed with an AND logic gate. Pyo reduction initiates induction of SoxR, a redox-sensitive transcriptional activator of SoxS. SoxR activates expression of a therapeutic molecule gene downstream to the SoxS promoter (PsoxS). The electronic control of the therapeutic molecule expression is carried out by an applied potential that induces Fcn reduction via interactions with the respiratory machinery.

Electrogenetic methods, devices, and systems utilizing redox biomolecules to transmit electronic information to engineered bacterial cells for transcriptional control in a synthetic gene circuit was described. The electrogenetic device described operates by utilizing electronic input to control the oxidation state of redox mediators, which, in turn, influence transcription in engineered bacterial cells, ultimately regulating biological output. The applied potential is a transduced input that interacts with redox mediators. The electronic induction is initiated by the oxidized (O) pyocyanin (Pyo) component, while reduced (R) or oxidized ferrocyanide (Fcn), interacting with respiratory machinery, allows electronic modulation of the induction level (Figure 5). The Fen (R/O) component plays a key role in this electronic control process. Overall, this electrogenetic device provides a means to electronically regulate gene expression in bacterial cells, enabling precise control of biological functions through electronic inputs (William et al., 2018; Table 1 patent 29).

An invention addressed challenges in gene therapy related to dosage sensitivity of therapeutic transgenes, where excessive or insufficient expression can lead to adverse effects. Viral-mediated gene transfer is effective, but high titers can result in overexpression toxicity. The inventors proposed a system termed “dosage-insensitivity,” involving a synthetic or non-mammalian microRNA (miRNA) construct to control transgene expression within a therapeutic window. This construct comprises a promoter, non-mammalian or synthetic miRNA expressed within an intron, a transgene, and miRNA binding sites for controlled expression. Unlike conventional mammalian-based miRNA constructs, the synthetic miRNA designs eliminate the risk of off-target effects. The system adapts to varying vector doses, downregulating transgene expression at high doses to maintain a stable level across cell populations. The invention introduces a fine-tuning mechanism, adjusting the number of miRNA binding sites and intron efficiency for optimal dosage-insensitivity. The genetic circuit construct presents a promising strategy for achieving controlled and stable transgene expression in gene therapy applications, particularly for genes requiring precise regulation to treat genetic conditions (Cobb et al., 2023; Table 1 patent 10).

The effectiveness of RNAi therapy can be improved with gene circuits and RNA delivery systems. An invention described a gene circuit that includes at least one RNA fragment capable of inhibiting gene expression and/or at least one targeting tag. This gene circuit can enrich in host organ tissues and self-assemble into a composite structure, contributing to the treatment of diseases by utilizing the RNA fragment to inhibit the expression of specific genes. The RNA fragment can consist of one or more medically significant RNA sequences, such as siRNA, shRNA, or miRNA sequences. This approach aims to provide a safe, precise, and efficient system for RNA delivery and gene circuitry in RNAi therapy (Chenyu et al., 2022; Table 1 patent 2).

Furthermore, an invention aimed to achieve the production of self-surface-releasing bacterial therapeutic colonies using a synthetic genetic circuit inserted into target probiotic bacteria. The method involves several steps: first, the genetic circuit incorporates sequences of an autotransporter protein from the genetic sequence of the target probiotic bacteria, a target protease responsive to a molecular signal in the target tissue, and the sequence of a target molecule for treatment or disease detection. Colonies activating the synthetic genetic circuit are selected by controlling the expression of the target probiotic bacteria, and those exhibiting self-surface release are then chosen as drug production colonies (Safak et al., 2023; Table 1 patent 5).

A genetic circuit involving synthetic minimal cells (SMCs) or a consortium of SMCs was designed for the production and delivery of therapeutic or diagnostic agents. The circuit comprises at least one sensor detecting specific conditions under which the agent is to be produced or delivered, a genetic circuit controlling the production of the agent upon sensor detection, and an outputting means for delivering the agent outside the SMC. The agent can be a protein, peptide, nucleic acid, or small molecule, and its production is regulated by transcription factors or a multi-enzyme biosynthetic pathway. The sensor, which can detect disease-related conditions, triggers the production or delivery of the agent. Methods for treating or diagnosing conditions using these SMCs, either individually or as a consortium, are also disclosed, offering targeted and responsive approaches for therapeutic or diagnostic purposes (Felix et al., 2022; Table 1 patent 7).

**FIGURE 6:**
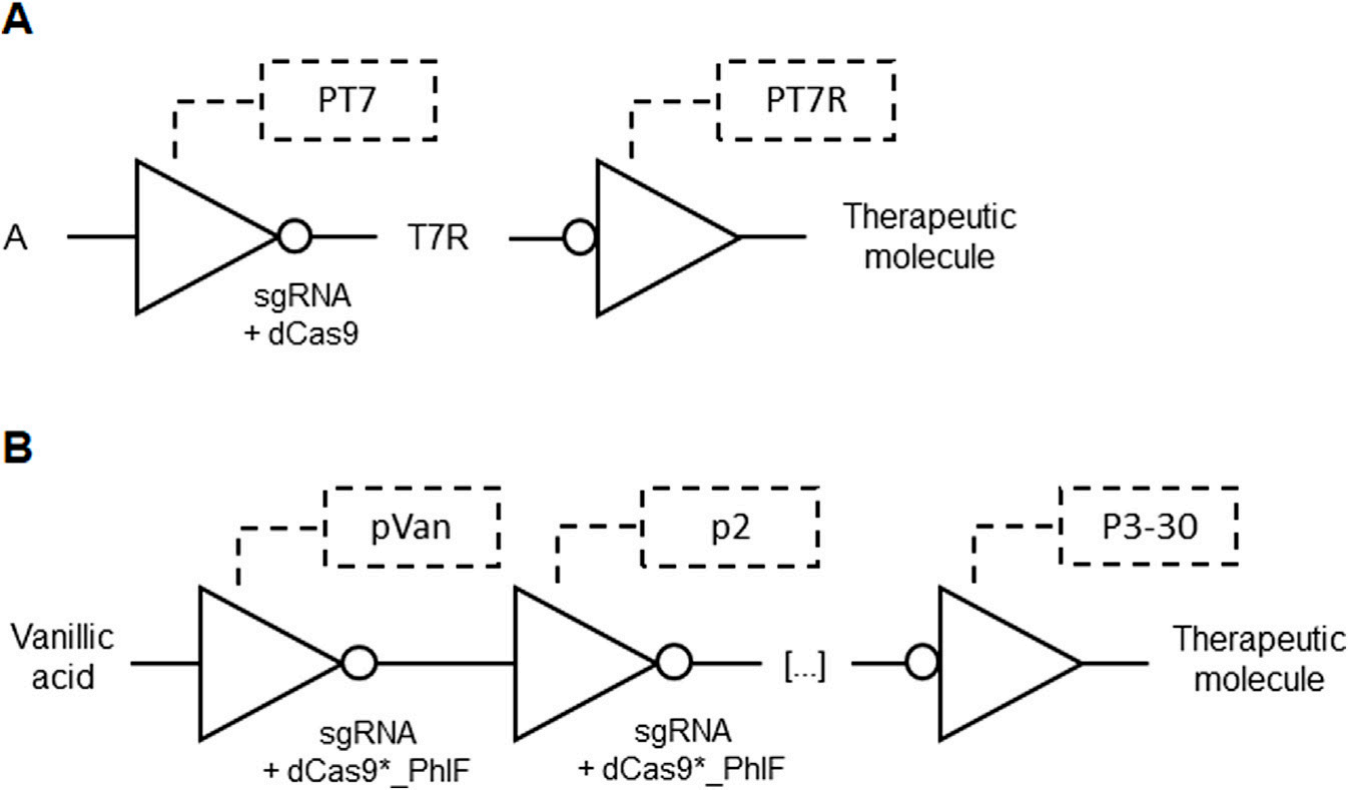
**(A)** Output expression of a therapeutic molecule by a cascade of NOT gates based on T7 promoter (PT7). An input A regulated by PT7 produce a sgRNA for dCas9 that suppress a T7 repressor (T7R) promoter (PT7R) upstream to a therapeutic molecule output. **(B)** Output expression of a therapeutic molecule by a cascade of NOT gates based on pVan and p2-30 promoters. Vanillic acid starts inducing the first sgRNA expression that combines with dCas9 fused with PhIF (dCas9*_PhIF) in a cascade of up to 30 connected NOT gates. The last promoter (p3-30) ending in a therapeutic molecule output is repressed.

Catalytically inactive RNA-guided endonucleases, such as dCas9 or dCpf1, were employed in genetic circuits to design complex cellular behaviors. These systems use guide RNAs to direct the inactive endonuclease to specific genetic loci in response to input signals, where it binds and inhibits transcription of target genes. This NOT gate inhibition can cascade into layered genetic circuits where transcribed output sequences act as new input signals, enabling sophisticated control of downstream genes in other logic gates. The circuits incorporate heterologous polymerases like T7 RNA polymerase (Figure 6A) to activate transcription across different species, enhancing versatility and functionality beyond the host organism’s native capabilities. This design allows precise modulation of gene expression, paving the way for advanced cellular engineering in therapeutic applications (Alec Andrew and Voigt Christopher, 2017; Table 1 patent 13).

To addresses dCas9 toxicity, which arises from its non-specific binding to numerous NGG PAM sites in the genome, an invention employed a fusion of dCas9 with the TetR-family PhlF repressor. This fusion specifically enhances PAM binding and reduces non-specific interactions, thereby eliminating toxicity and allowing high expression levels without harming cell health. By incorporating PhlF operators and sgRNA targeting sequences in promoters, the invention creates CRISPR/dCas9-based logic gates with improved cooperativity and non-linear response curves. Enhanced cooperativity was achieved with a set of 30 sgRNAs designed as NOT gates (Figure 6B). These gates enable the development of complex genetic circuits with reduced toxicity, facilitating the scaling up of genetic circuits by regulating output sequence expression through the introduction of these novel logic gates into cells (Christopher and Shuyi, 2018; Table 1 patent 14).

A sophisticated system was proposed for controlling gene expression in modified cells using multivalent cooperative transcription factor assembly. This system employs synthetic transcription factors (sTFs) that bind to tandem DNA binding motifs (DBMs) located upstream of a promoter, which can either activate or repress the transcription of a gene of interest (GOI). A key component is the molecular clamp (MC), a protein with multiple ligand binding domains (LBDs) that tether together the sTFs, enhancing their cooperative binding to DBMs and thereby finely tuning gene expression. This modular approach allows precise control over gene expression by adjusting the number and strength of sTFs and their interactions with the DBMs and LBDs. The system enables both “ON” and “OFF” switches for gene expression, depending on whether the sTFs carry transcription activator (TA) or transcription repressor (TR) domains, and can be controlled by multiple input signals for more complex regulation, making it highly useful for applications like therapeutic cell engineering and gene therapy (Khalil Ahmad et al., 2023; Table 1 patent 23).

A RNA-based genetic circuit that can modulate gene expression in response to specific RNA triggers was developed using RNA-editing enzymes known as adenosine deaminases acting on RNA (ADARs) to detect target RNA sequences through base pairing, leading to a specific editing event that removes premature stop codons in the sensor mRNA. This editing converts a stop codon (UAG) into a tryptophan codon (UGG), allowing translation to proceed and produce a desired protein output. The circuit can function with endogenous ADAR levels or through the overexpression of exogenous ADAR. The system’s flexibility allows it to be applied in various therapeutic contexts, potentially enabling precise, cell-specific treatments with minimal off-target effects (Collins James et al., 2014; Table 1 patent 24).

A red light-regulated genetic circuit system (REDLIP) was proposed for mammalian cells, offering precise, non-toxic, and efficient control of gene expression. The REDLIP system includes a red light-sensitive protein (660 nm) derived from bacteria, plants, or fungi, fused with a DNA-binding domain protein (Gal4) and a transcription activator element (LDB3 nanochaperone protein). This combination allows for controlled gene expression by red light, with the ability to switch off using far-red light (780 nm). The system’s gene expression can be precisely regulated by adjusting light intensity and duration, providing a highly controllable tool for basic research, regenerative medicine, and gene therapy (Haifeng et al., 2023; Table 1 patent 25).

For timed delivery of therapeutic proteins, an invention described synthetic genetic circuits engineered into cells to harness their internal circadian rhythms. This system uses promoters, enhancers, and repressors from circadian-responsive genes to regulate the expression of therapeutic genes in alignment with the body’s natural biological clock. By doing so, these engineered cells can deliver treatments at optimal times of day, enhancing their efficacy and minimizing side effects. This technology aims to improve the treatment of various conditions, including inflammatory diseases, cancer, and metabolic disorders, by synchronizing drug delivery with the body’s circadian rhythms, potentially eliminating the need for inconvenient and frequent dosing schedules (Farshid et al., 2022; Table 2 patent 5).

Cells engineered with genetic circuits were developed to detect mechanical stimuli and subsequently produce therapeutic proteins. These circuits involve promoters from mechanically responsive genes, such as NF-κB or PTGS2, linked to genes encoding therapeutic proteins. When mechanical forces such as tension, compression, or shear stress are detected, these promoters activate, driving the expression of the therapeutic gene. For example, the TRPV4 ion channel can sense mechanical loading, triggering intracellular signaling cascades that result in the production of therapeutic agents like IL-1 receptor antagonist (IL-1Ra). These engineered cells can be used to treat a variety of conditions, including inflammatory diseases, cancer, and cardiovascular diseases, by delivering therapeutic proteins in response to mechanical stimuli, thereby synchronizing treatment with the body’s natural mechanical environment (Farshid et al., 2023; Table 2 patent 6).

A novel gene therapy approach was proposed for using CRISPR activation to treat neurological disorders like epilepsy by upregulating endogenous genes. This method involves using single guide RNAs (sgRNAs) that target specific regulatory sequences of endogenous human genes associated with the disorder. The sgRNAs form a complex with a deactivated CRISPR nuclease (dCas9) and a transcriptional activator, which then binds to the regulatory sequence of the target gene, enhancing its expression. Unlike traditional gene therapies that introduce exogenous genes, this approach leverages the body’s own genes for therapeutic effects, allowing for more precise and graded upregulation of gene expression. This method also supports combinatorial gene therapy by simultaneously targeting multiple genes, potentially offering a more effective treatment for epilepsy and other neurological conditions by restoring normal brain function through multiple pathways. The invention aims to overcome limitations of current gene therapies, such as size constraints of viral vectors and lack of normal mRNA splicing, providing a safer and more versatile treatment option (Gabriele et al., 2023; Table 2 patent 7).

**FIGURE 7:**
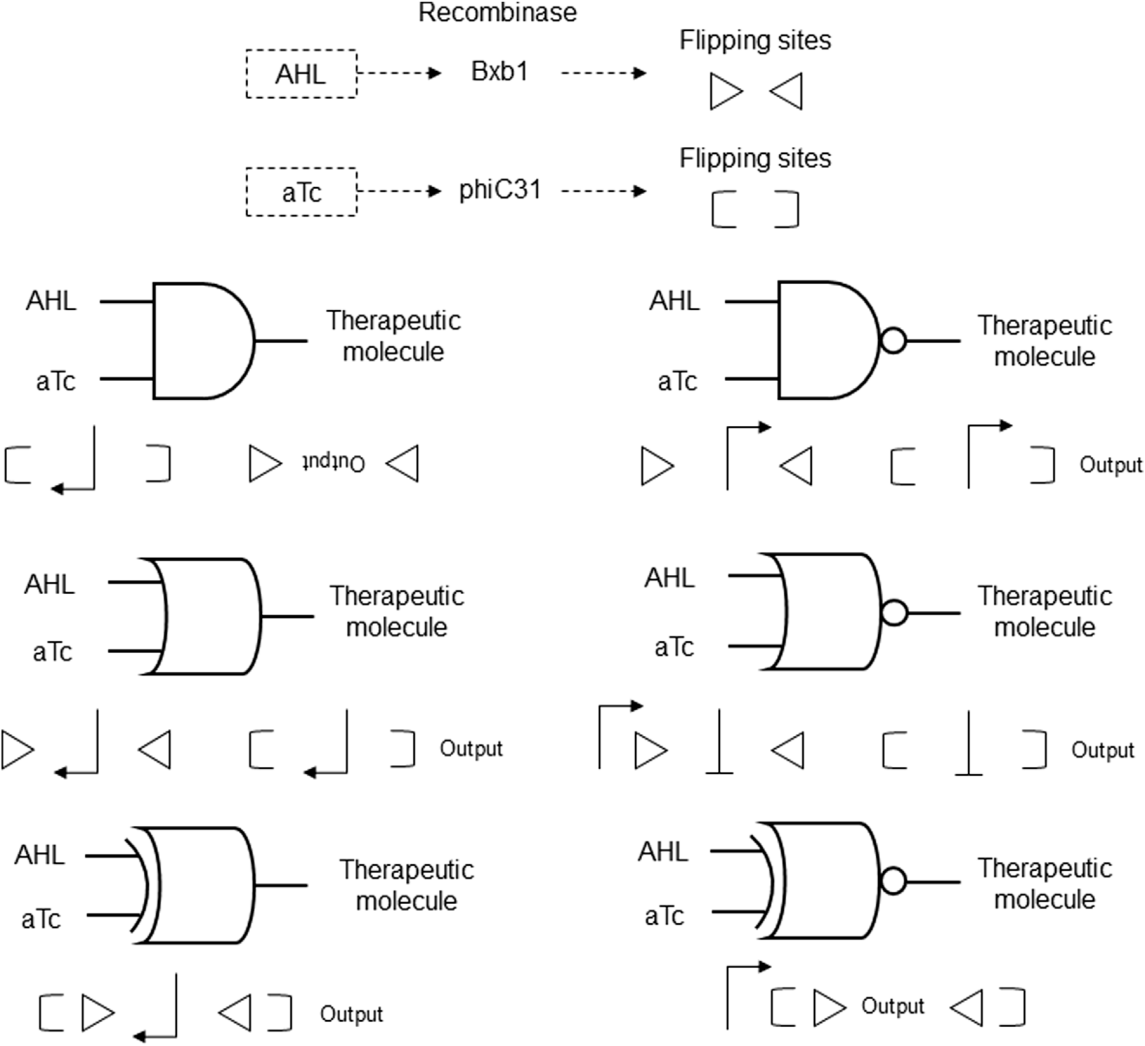
Recombinase-based genetic circuits with AND, OR, NOR, NAND, XOR, and XNOR logic gates. The inputs AHL and aTc induce expression of Bxb1 and phiC31 recombinases that flip oriented sites represented by triangles and parentheses, respectively. The positions and orientations of promoters (sharp arrows) and output with the positions and orientations of flipping sites for each recombinase are shown. Blunt arrows indicate inhibition by 5′ to 3′ orientation.

A modular DNA assembly strategy was created with synthetic recombinase-based systems that integrate logic functions with DNA-based memory in living cells. These genetic circuits employ the chemical inducers N-acyl homoserine lactone (AHL) and anhydrotetracycline (aTc) to activate the specific recombinases Bxb1 or phiC31 via inducible promoters. These recombinases then target flanking recognition sites to invert or excise DNA segments, conditionally regulating gene expression. The genetic circuits are designed to include multiple logic gates, such as AND, OR, NOR, NAND, XOR, and XNOR, constructed from sequences encoding promoters and recombinases, flanked by recognition sites that enable recombinase-mediated regulation of gene expression (Figure 7). The logic gates can perform a wide range of two-input Boolean functions, with the ability to maintain memory over multiple cellular generations, thus providing a robust platform for therapeutic applications (Timothy and Piro, 2014; Table 2 patent 8).

### Treatment of infectious diseases

Lytic treatments to bacterial infections can release harmful endotoxins. A genetic circuit was designed to operate through engineered bacterial phagemids designed to address bacterial infections with reduced side effects. These phagemids carry a synthetic genetic circuit that includes sequences encoding non-lytic antimicrobial peptides (AMPs) and antibacterial toxins, an origin of replication, and a bacteriophage-packaging signal. The phagemids cause non-lytic bacterial death since they lack genes necessary for assembling complete bacteriophage particles, minimizing the risk of harmful endotoxin release. *In vivo* testing demonstrates their efficacy in a murine peritonitis infection model. The administration of recombinant phagemids or their particles presents a non-antibiotic alternative for treating bacterial infections while avoiding issues associated with lytic and replicative bacteriophage use (Russell-John et al., 2022; Table 1 patent 1).

**FIGURE 8:**
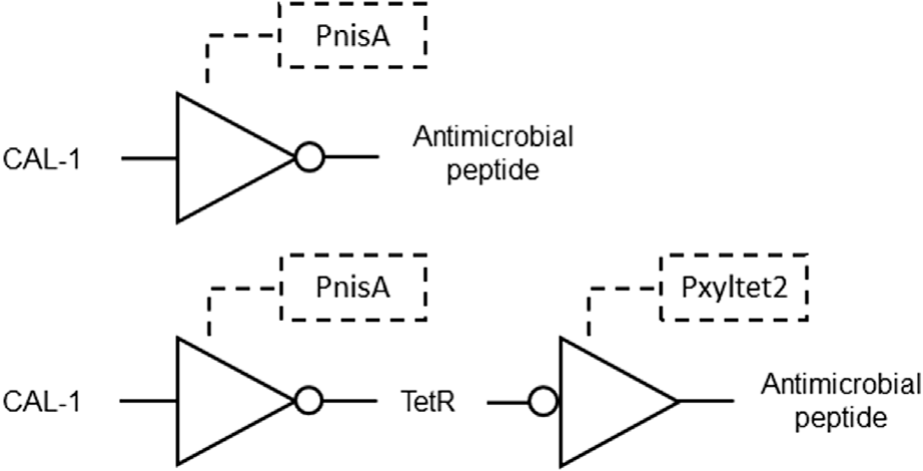
Gene circuit for detection and treatment of *Vibrio cholera* formed by NOT gates. Top: CAL-1 is recognized by the CqsS domain of the hybrid receptor, inducing NisA phosphorylation by the NisK domain, which repress PnisA and activates a downstream antimicrobial peptide. Bottom: CAL-1 repress PnisA and activates downstream TetR expression. TetR inhibits Pxyltet2 and promotes expression of an antimicrobial peptide.

Another invention describes non-naturally occurring bacteria engineered to detect the CAI-1 molecule produced by *Vibrio cholerae*. These bacteria have a hybrid receptor consisting of a CqsS ligand binding domain and a NisK histidine kinase domain. A genetic circuit is integrated into the bacteria, including promoters responsive to CAI-1 and the histidine kinase, controlling the expression of output molecules. In some embodiments, the output molecules include antimicrobial peptides or reporter polypeptides (Figure 8). This system allows the engineered microorganisms, such as *Lactococcus lactis*, to rapidly respond to *V. cholerae* infection by expressing specific modules that inhibit or kill the pathogen, or provide a colorimetric signal indicating detection. Methods are disclosed for administering these bacteria to subjects at risk of cholera infection, enabling detection and potential treatment by triggering the expression of therapeutic molecules. The approach offers a versatile tool for detecting and combating cholera infections, especially in outbreak scenarios, with potential applications in diagnostics and therapeutics (Mao et al., 2020; Table 1 patent 27).

A genetic circuit was developed aiming at treating multi-drug-resistant tuberculosis (MDR-TB) by modulating the activation of the prodrug ethionamide. The mycobacterial monooxigenase ethA catalyzes activation of ethionamide but is transcriptionally repressed by EthR. The circuit involves a pharmaceutical composition that includes a compound preventing the EthR protein from binding to the ethA promoter, thus enhancing the expression of ethA. The system utilizes a synthetic gene network where EthR is fused with the VP16 transactivation domain, which activates transcription from a minimal promoter when bound to its operator. In the presence of a cell-permeable, non-cytotoxic inducer, the binding of EthR-VP16 is inhibited, resulting in the repression of the target gene. This approach aims to increase the sensitivity of MDR-TB to ethionamide, allowing for lower, less toxic dosages of the drug to be used effectively (Martin et al., 2010; Table 2 patent 2).

### Treatment of cancer

**FIGURE 9:**
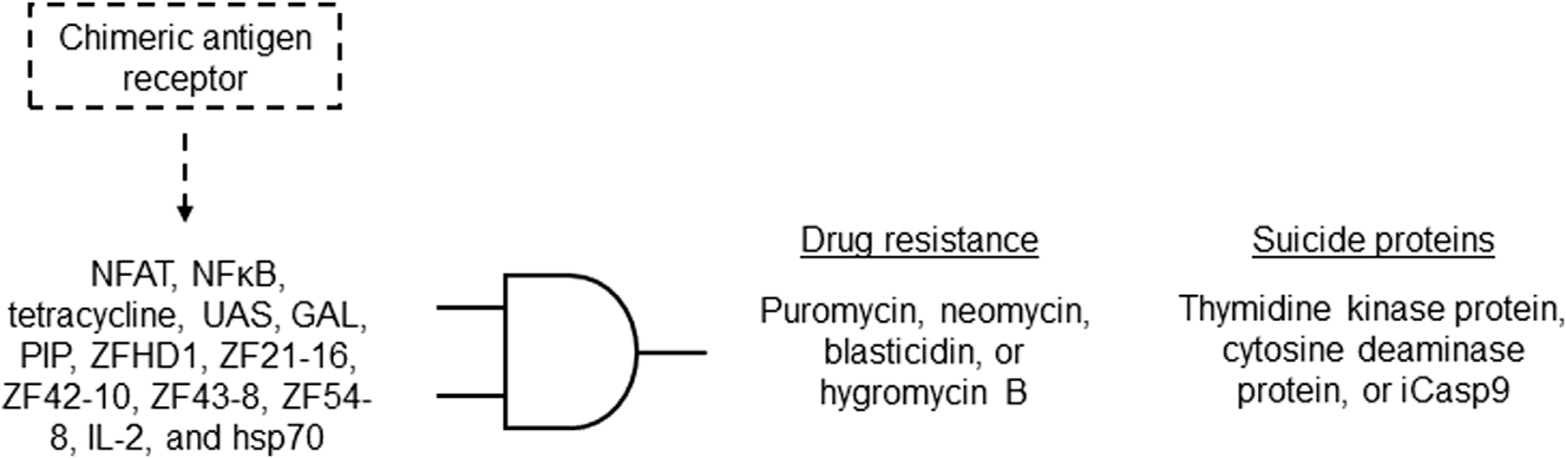
Representation of the possible gene circuits for the proposed CAR-T cell technology formed by an AND gate. The CAR library activates the expression of at least one of the cis-acting and transcription factors shown, by which their combination leads to the expression of drug resistance and/or suicide proteins.

Challenges in chimeric antigen receptor (CAR)-T cell technology were addressed by proposing a CAR cell library with individualized properties, aiming to overcome issues such as drug resistance in CAR-T cells and the inability to target unknown antigens. This invention provides a carrier assembly carrying three genetic elements: multiple first genetic elements encoding a chimeric antigen receptor library, a second gene element containing various unique gene circuits, and a third gene element encoding unique inducible proteins. The gene circuit is preprogrammed to regulate the expression of the inducible protein when the encoded chimeric antigen receptor is activated. The gene circuits include regulatory cis-acting and transcription factors, offering flexibility in design. The inducible proteins include drug resistance and/or suicide proteins that can induce cell death when expressed (Figure 9). This invention introduces a comprehensive approach for preparing and constructing a chimeric antigen receptor cell library, along with screening methods for *in vivo* and *in vitro* antigens, with potential applications in addressing disease antigen heterogeneity, variability, and evolution (Shi and Wenyan, 2021; Table 1 patent 28).

**FIGURE 10:**
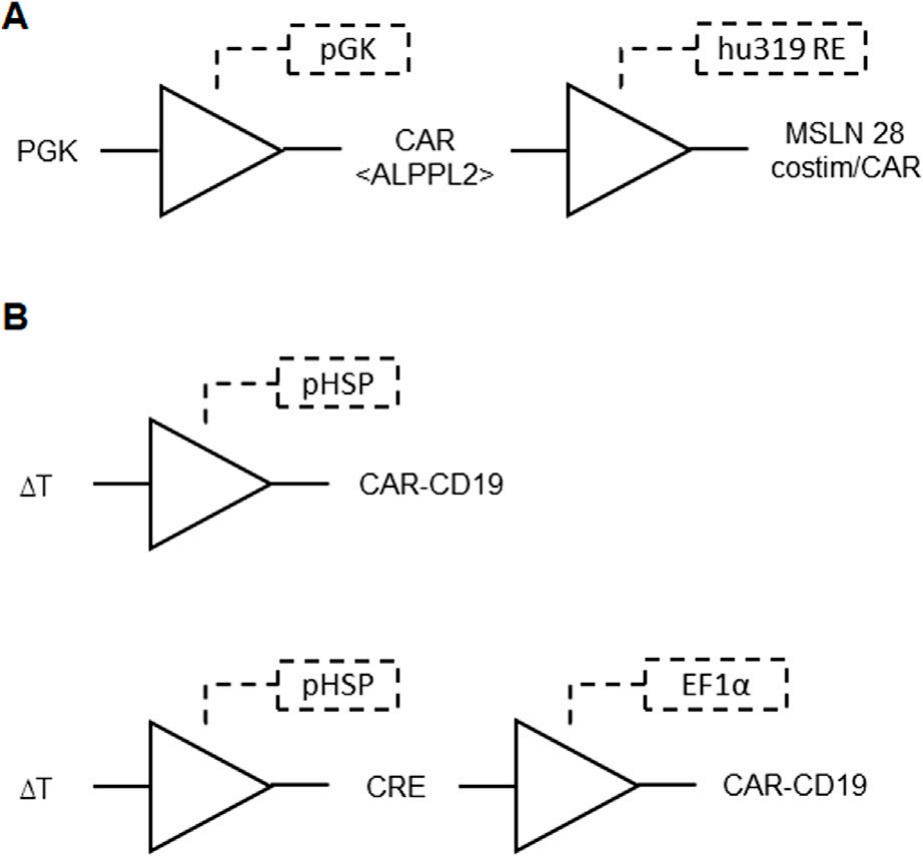
**(A)** Genetic circuit for controlled CAR expression formed by BUFFER gates. The ALPPL2 CAR is constitutively expressed by pGK promoter. CAR binding to ALPPL2 triggers hu319 RE induced expression of mesothelin (MSLN) costim only or full CARs. These constructs feature a CAR with only the costimulatory domain and a construct that includes a full CAR with CD28 costimulatory molecule and CD3z. **(B)** Temperature responsive gene circuit for the proposed CAR-T cell technology formed by BUFFER gates. Top: temperature changes are input signals for the heat shock protein promoter (pHSP), leading to downstream expression of a CAR-CD19 gene. Bottom: temperature changes induce expression of CRE recombinase, which catalyzes recombination of a DNA segment with two CRE responsive elements that place the CAR-CD19 gene downstream to the constitutive promoter EF1α, leading to expression of CAR-CD19.

Aiming to minimize side effects and optimize therapeutic efficacy, another invention introduced genetic circuits that enable precise regulation of CAR-T cell activity. These circuits utilize transcriptional regulatory regions that activate customizable genetic programs in T-cells upon CAR engagement with target antigens. The genetic circuits described involve constructs using the hu319 response element and Myc-ALPPL2 BB CAR to target and destroy specific cancer cells. This CAR is constitutively expressed in T cells, which, upon binding to the ALPPL2 antigen on target cells, activate the T cells and trigger the CAR payload with mesothelin costim-only CAR or mesothelin full CAR with CD28 and CD3z (Figure 10A). This payload enables the T cells to further target and eliminate cells expressing mesothelin, ensuring controlled CAR expression, preventing antigen-independent signaling and T-cell exhaustion, while enhancing anti-tumor responses and enabling fine-tuned therapeutic gene expression. The circuits can be applied *in vivo* and *in vitro*, with potential uses in T-cell activity reporting and delivering therapeutic payloads in a highly controlled manner (Roybal et al., 2023; Table 1 patent 21).

An invention addressed the challenges associated with the targeted application of genetically engineered T cells in therapeutic contexts, particularly in the treatment of solid tumors. Unlike small molecules and biotherapeutics, T cells possess intrinsic capabilities for targeted and sustained disease treatment due to their ability to detect and respond to environmental cues. The invention focuses on enhancing the control over T cell activity by introducing a genetic circuit that responds to external signals, specifically temperature changes induced by techniques such as focused ultrasound and magnetic hyperthermia (Figure 10B). This spatiotemporal control aims to mitigate off-target toxicity in solid tumor applications, where existing strategies often fall short. The genetic architecture involves inducible promoters, transactivator genes, and payload genes, allowing for precise modulation of T cell activity in response to thermal stimulation and immune cell signals (Abedi Mohamed et al., 2023; Table 1 patent 11).

**FIGURE 11:**
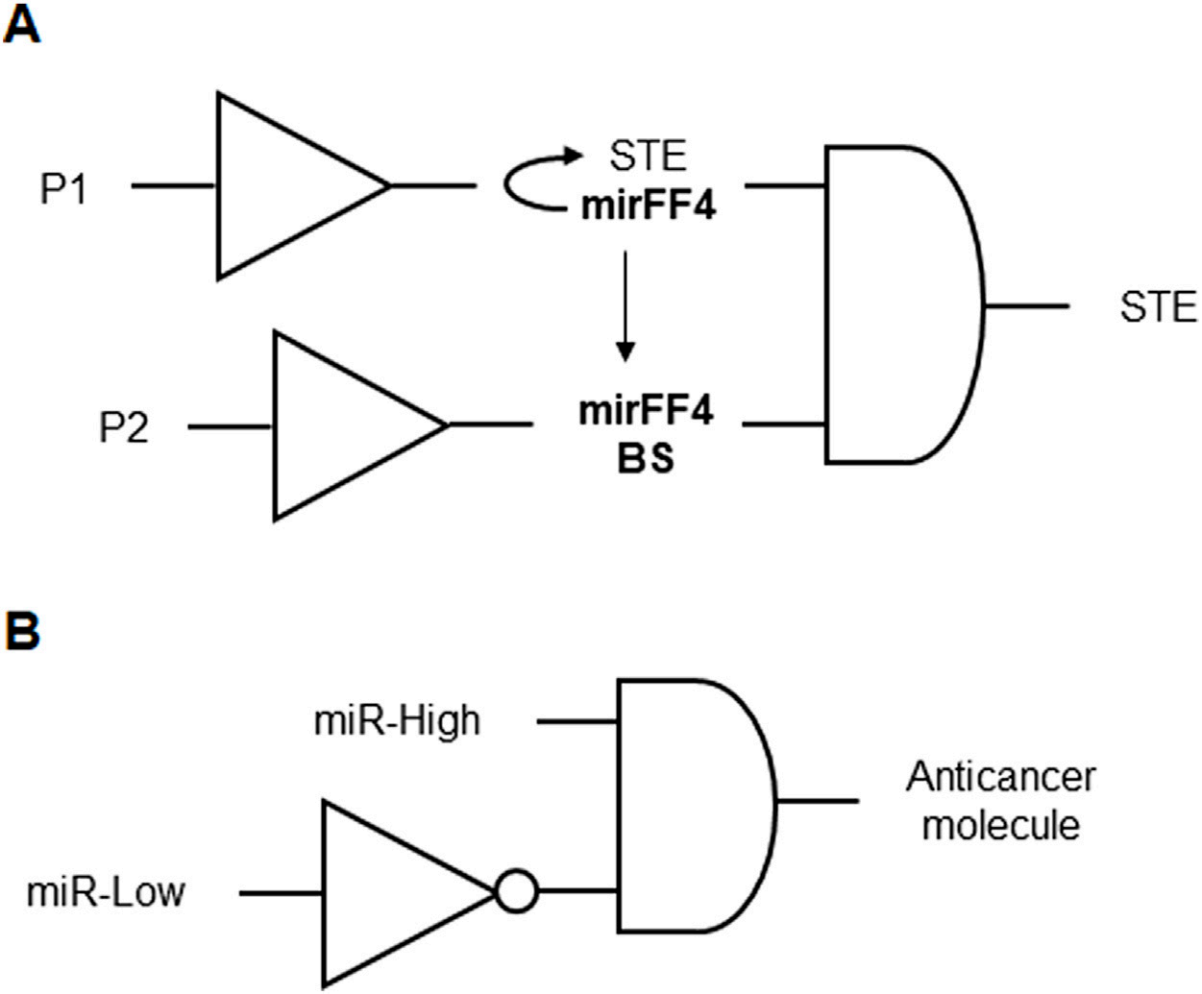
**(A)** RNA-based genetic circuit for tumor immunotherapy formed by two BUFFERs connected by an AND gate. The input P1 induces the expression of the surface T-cell engager (STE) and the synthetic miRNA mirFF4 that forms a loop to constantly degrade the STE mRNA. When the input P2 is present, multiple mirFF4 binding sites (mirFF4 BS) are expressed, diverging mirFF4 and granting the output STE expression. **(B)** Representation of the genetic circuits for the proposed microRNA detection device formed by a NOT gate connected to an AND gate. miR-Low represents an input of a low level of one or more endogenous microRNAs. miR-High represents an input of a high level of a single microRNA. The anticancer molecule is regulated by a promoter that is both activated by miR-High and inhibited by the repressor induced by miR-Low in the inverter. The anticancer molecule output is expressed only when miR-High is present and miR-Low is absent in the AND gate.

RNA-based genetic circuits were engineered to produce cell-specific therapeutic outputs, such as T cell engagers that trigger cytotoxic T cell responses against tumors. These genetic circuits feature RNA-based logic gates that are selectively activated in cancer cells, leading to the expression of immunomodulatory molecules (Figure 11A). This platform includes mechanisms for tunable multi-output therapy, employing cytokines, chemokines, and immune checkpoint inhibitors to enhance immune responses. The circuits are designed to achieve localized treatment upon systemic administration, ensuring safety and efficacy. By engaging T-cells and utilizing combinatorial therapy outputs, the system aims to eradicate tumors and prevent relapses through immune memory formation (Lu and Mimee, 2017; Table 1 patent 22).

Another genetic circuit was described involving engineering macrophages to become heat-inducible and secrete therapeutic payloads specifically within the tumor microenvironment to counteract the immunosuppressive effects of tumor-associated macrophages (TAMs). These macrophages are designed with a first inducible promoter linked to a recombinase gene that activates upon thermal stimulation, generating recombinase, which then catalyzes a recombination event. Like in Figure 9, this recombination event either inverts or removes a sequence flanked by recombinase target sites, enabling a second constitutive promoter to induce transcription of a payload gene that was previously inactive. This setup ensures that output therapeutic molecules such as pro-inflammatory cytokines or pro-death proteins are released exclusively within the tumor, minimizing systemic side effects (Farooq Abdullah et al., 2023; Table 1 patent 19).

Genetic circuits were proposed for engineering of megakaryocytes and platelets to target and destroy circulating tumor cells (CTCs). The gene circuit constructs include a promoter linked to c-MYC, BMI1, BCL-XL, and a recombination site, enabling the expression of engineered antibody sequences. These antibodies contain split toxin sequences flanked by intein fragments, which can be reassembled inside target cells to form a functional toxin, inducing cell death. Engineered platelets derived from these megakaryocytes can carry these antibodies, targeting CTCs in the bloodstream to prevent metastasis, offering a potentially adaptive and less systemic method for treating metastatic cancer (Tara, D. 2022; Table 1 patent 20).

Genetic circuits and cell state classifiers were designed for detecting the miRNA profile of a cell for applications in therapeutics or diagnostics. These classifiers integrate multiple genetic circuits, using transcriptional or translational control, to detect the microRNA profile through engineered downregulation of an output molecule’s expression by specific miRNA. By incorporating target sites for the miRNA into genetic circuits controlling the output molecule expression, the system can sense multiple inputs simultaneously. The classifiers are utilized for detecting diseased cells, such as cancer cells, with options for diagnostic applications involving detectable output molecules like fluorescent proteins. Furthermore, the genetic circuits can be designed to express therapeutic molecules for treating diseases like cancer. The described genetic circuit serves as a cell state classifier, implementing a logic circuit composed of a NOT gate connected to an AND gate. This circuit aims to produce a high level of an output molecule, when miR-Low is low and miR-High is high (Figure 11B). The classifiers exhibit versatility and specificity in detecting various cellular states, making them valuable tools for diagnostics and therapeutics, and can be employed in diverse cell types, such as human cancer cells (Jin Hang and Ron, 2019; Table 1 patent 9).

Synthetic RNA circuits were designed for sophisticated gene regulation without relying on DNA. These circuits use mRNA for gene transfer, providing safety advantages by avoiding genomic integration. They leverage RNA-binding proteins (RBPs) to create complex and scalable post-transcriptional control mechanisms. These circuits use two RNA molecules: one containing sequences recognized by cell-specific microRNAs and encoding a repressor protein, and another containing sequences targeted by different microRNAs and encoding an output protein. In the target cells, the specific microRNAs suppress the repressor, allowing the production of therapeutic or functional proteins. These RNA molecules can be encoded on replicons or plasmids, enabling applications such as cancer treatment by exploiting cell-specific microRNA profiles (Ron et al., 2022; Table 1 patent 15).

**FIGURE 12:**
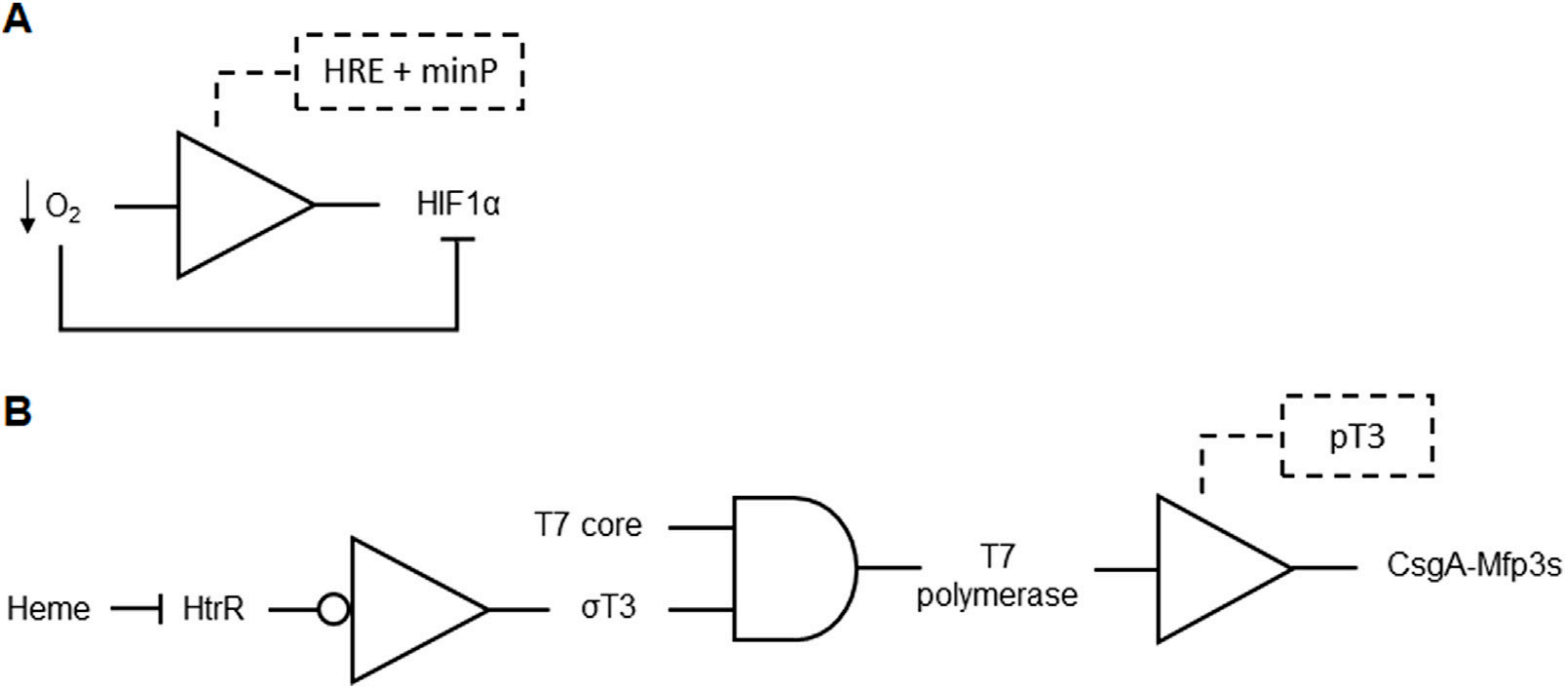
**(A)** Positive feedback formed by a BUFFER gate restoring HIF1α under hypoxic conditions. While a low O_2_ tumor environment reduces HIF1α levels, the BUFFER formed by the promoters HRE and minP senses the low O_2_ level and responds with HIF1α expression in a positive feedback. **(B)** Blood-sensing genetic circuit for wound repair formed by coupled NOT, AND and BUFFER gates. ChuA is constitutively expressed to deliver heme to the heme-sensitive transcriptional repressor (HtrR). Heme inhibits HtrR, inducing expression of the σT3 fragment. The constitutively expressed T7 core and the induced σT3 fragment forms the T7 polymerase that tightly binds to T3 promoter (pT3), inducing expression and secretion of the adhesive protein components CsgA-Mfp3s.

A DNA-based hypoxia biosensors was developed to detect and respond to low oxygen levels in tissues, particularly in tumor microenvironments where hypoxia is prevalent. These biosensors utilize a hypoxia-inducible promoter, which can include elements like Egr-1 binding sites, metal-response elements (MRE), and hypoxia-response elements (HRE). They also include sequences encoding functional elements (e.g., reporter genes like luciferase or fluorophores) and feedback elements (e.g., HIF1α or HIF2α for positive feedback). By incorporating these HREs and promoters, the biosensor ensures that the gene of interest is expressed under hypoxic conditions (Figure 12A). Additional engineered proteins may further modulate gene expression, enhancing the biosensor’s sensitivity and robustness against tumor-related hypoxia dysregulation. The system can include various combinations of these elements to improve detection and imaging of hypoxia *in vivo*, thus enhancing targeted therapies for hypoxic tumor environments (Yannick et al., 2024; Table 1 patent 16).

A thermally inducible genetic system composed by a promoter responsive to temperature changes that drives the expression of a recombinase gene was proposed. Upon thermal stimulation above physiological temperature, the expressed recombinase enzyme catalyzes a recombination event that links a second promoter to a payload gene, which was previously unlinked, enabling the second promoter to drive the transcription and subsequent translation of the payload gene. The recombination event can involve sequence inversion or removal and is designed to be irreversible. This system was designed for expression by engineered *Escherichia coli* cells. The temperature-sensitive transcription factors, which repress or activate the first promoter based on temperature, ensure precise control of the recombinase expression and subsequent payload gene activation. The payload genes can encode various proteins, including therapeutic agents to treat cancer (Abidi et al., 2022; Table 1 patent 17).

A genetic circuit was proposed for oncolytic adenovirus regulation that employs a cell-specific promoter as a master switch to control the expression of a transcriptional activator, which in turn regulates the adenovirus E1A gene essential for viral replication. In this system, microRNA target sequences serve as secondary switches to respond to microenvironmental cues, enhancing the specificity and efficiency of gene expression while reducing the risk of leakage. By removing the E1B and E3 genes, the adenovirus is rendered less toxic to normal cells and gains increased packaging capacity. The described genetic circuit can be rapidly assembled, facilitating the integration of multiple therapeutic genes into the adenovirus. It aims to enhance the safety, specificity, and therapeutic efficacy of oncolytic adenoviruses, offering a robust platform for cancer treatment (Zhen et al., 2018; Table 2 patent 4).

A gene system was designed to specifically recognize and target P53 mutations, which are prevalent in many human cancers. The system employs the CRISPR-Cas9 mechanism, driven by a P53-binding enhancer region combined with a minimal-SV40 promoter, to express sgRNA and Cas9 proteins in cells containing wild-type P53. In cells with wild-type P53, the CRISPR-Cas9 system inhibits diphtheria toxin (DT) expression, protecting these cells from apoptosis. In contrast, P53-deficient cells fail to express the CRISPR-Cas9 system effectively, allowing DT expression and inducing cell death. This selective expression mechanism ensures that only P53-deficient cancer cells are targeted and killed, while normal cells are spared (Weiren et al., 2022; Table 2 patent 9).

### Treatment of bleeding

Engineered living biofilm glues designed with a genetic circuit for autonomous on-demand mechanical repairs was proposed. The system comprises two bacterial strains embedded in a biofilm linked by an inducible cell-cell communicating genetic circuit. The glue-producing strain secretes a signal molecule and expresses a fusion protein triggered by an environmental inducer, while the adhesion-enhancing strain expresses tyrosinase induced by the signal molecule. The environmental inducer includes heme, and the genetic circuit is responsive to signals such as blood/heme. Upon sensing blood from a wound, the strains localize to the damaged site, communicate via a cell-cell network, and repair the defect with amyloid glue components. The blood-induced glue system is constructed with a gene network involving the heme transporter (ChuA membrane protein) and heme-sensitive transcriptional repressor HtrR39 (Figure 12B). The system responds to blood concentrations as low as 10 parts per million, demonstrating its sensitivity and potential for autonomous repairs in various settings (Chao et al., 2023; Table 1 patent 3).

Another invention describes engineered microorganisms, such as *E. coli*, functionalized with synthetic genetic circuits to detect gastrointestinal (GI) bleeding and record such events in cellular memory. These microorganisms are engineered to express a heme-responsive transcription factor, such as HrtR from *L. lactis*, and a heme transporter, like ChuA, which allow the detection of heme in the GI tract. The genetic circuits include promoters responsive to the heme-responsive transcription factor, enabling the production of reporter molecules or therapeutic proteins in response to heme detection. They can be administered orally, potentially in pill form, and later analyzed either *in vivo* or in stool samples to detect bleeding and assess treatment needs (Lu et al., 2017; Table 1 patent 18).

### Treatment of metabolic disorders

**FIGURE 13:**
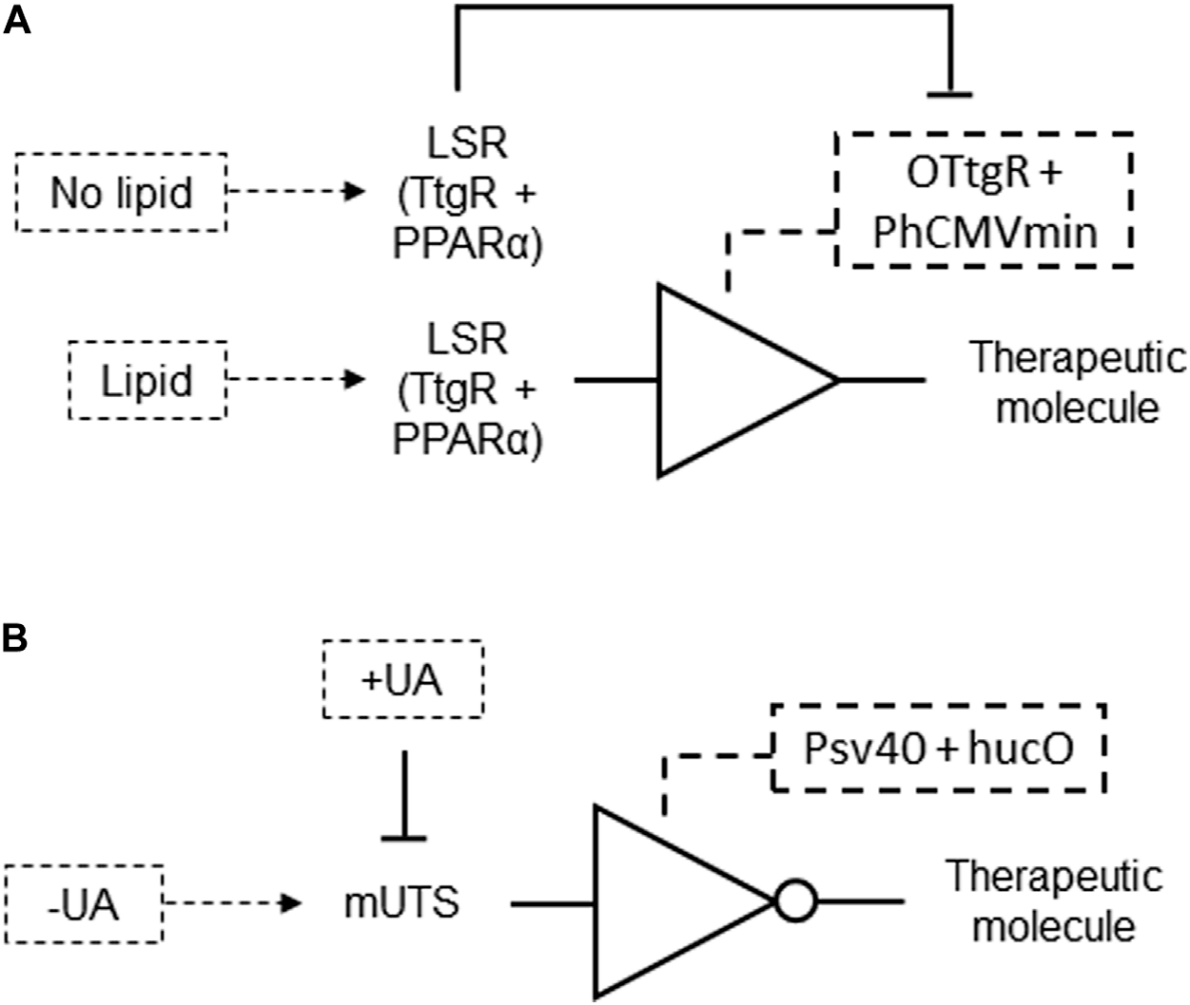
**(A)** A lipid responsive genetic circuit formed by a BUFFER gate. Lipid levels induce LSR association with an activation complex, which binds to the phloretin-responsive repressor (TtgR)-specific operator OTtgR linked to a minimal promoter, to produce a therapeutic molecule output to treat diet-induced obesity. LSR associates with an inhibitory complex and repress the therapeutic molecule expression in the absence of fatty acids. **(B)** Uric acid homeostasis regulator formed by a NOT gate genetic circuit. While absence of uric acid (UA) allows mUTS binding to hucO and repression of output, the presence of UA inhibits and releases mUTS from hucO, resulting in the production of a therapeutic molecule output.

A genetic circuit was developed involving a mammalian cell engineered to control dietary energy homeostasis by responding to lipid levels. This system features an intracellular lipid-sensing receptor (LSR), a fusion protein combining a phloretin-responsive repressor (TtgR) and the human peroxisome proliferator-activated receptor alpha (PPARα). LSR binds to the specific operator OTtgR linked to the minimal promoter PhCMVmin to control transgene expression. In the absence of fatty acids, the LSR represses transgene expression by associating with an inhibitory complex. When fatty acids are present, the LSR switches to an activation complex, fully inducing transgene expression (Figure 13A; Table 2 patent 1). This gene circuit can detect the presence of fatty acids and accordingly switch on or off the expression of specific genes, which could be a protein that helps reduce fat absorption or signals satiety. It aims to provide a novel strategy for treating diet-induced obesity by modulating caloric intake in response to lipid levels (Katrin and Martin, 2014; Table 2 patent 1).

Another genetic circuit was designed for mammalian cells to regulate gene expression in response to uric acid levels. This system utilizes vectors encoding a mammalian urate transsilencer (mUTS) formed by a bacterial uric acid sensor-regulator (HucR) fused to the human Kruppel-associated box-protein (KRAB), and an operator sequence (hucO) that specifically binds this sensor-regulator. In the absence of uric acid, HucR binds to the hucO operator, repressing the expression of a transgene. When uric acid is present, it triggers the release of HucR from hucO, allowing the expression of a gene under the control of a minimal promoter (Figure 13B; Table 2 patent 3). This gene could encode an endogenous or exogenous protein involved in uric acid metabolism or detection. The invention provides a method for detecting and degrading excess uric acid, offering a potential therapeutic approach for conditions like gout and hyperuricemia by utilizing genetically engineered mammalian cells housed in nano- or microcontainers (Christian et al., 2010; Table 2 patent 3).

## Conclusion and future perspectives

Synthetic gene circuits can be customized for precise control of therapeutic interventions. This may lead to personalized gene therapies that respond specifically to individual patient needs, enhancing treatment efficacy and minimizing side effects. As highly sensitive biosensors, they may provide real-time therapy by accurate monitoring various biomarkers or pathogens and responding with the synthesis of a therapeutic molecule. The intricate maintenance of physiological homeostasis correlates with the sensing and self-regulating ability of gene circuits. This way, synthetic gene circuits can be developed to interact with physiological signals and respond by restoring a homeostasis process unbalanced by pathological conditions (Ye and Fussenegger, 2014).

In tissue engineering applications, synthetic gene circuits may lead to the development of advanced regenerative therapies, where cells are programmed to respond dynamically to their environment for optimal tissue repair. Implantable devices comprised of engineered tissues or cells encapsulated in biomaterials containing synthetic gene circuits can be promising new technologies. They can be customizable tolls and sophisticated biological input sensing systems that produce on-demand output bioactive molecules to treat or prevent pathologies, restore health conditions, or even improve biological features (Sedlmayer et al., 2018).

Genetic circuits offer several advantages and disadvantages. Genetic circuits must be adjusted to fulfill the specific requirements of a given application, but developments in libraries of well-defined components and advanced computational tools have simplified the process of designing and fine-tuning genetic circuits. While individual transcription factors may seem nontoxic, combining multiple regulators can often result in acute toxicity. In addition, homologous recombination can compromise circuit integrity, and using recombinases for circuit design may lead to irreversible states and mixed populations. The context dependency of genetic parts can alter circuit behavior when components are reorganized, causing cross-talk and circuit failure. Resource overuse, retroactivity, and environmental variability also pose significant challenges, affecting circuit functionality and stability. The designability of CRISPR systems allows the construction of large, complex circuits. However, predicting guide RNA orthogonality is challenging, and CRISPR circuits often suffer from toxicity due to nonspecific Cas9 binding. Improved understanding of these issues and learning from natural systems can help develop more robust design rules for synthetic gene circuits (Brophy and Voigt, 2014).

Synthetic gene circuits has generated a large number of journal articles. Most of them remains at the publication stage, with few advancing to patent applications and achieving commercial success. Nevertheless, the ability to create gene therapies in programmable platforms and monitor biomarkers in real-time can revolutionize the possibilities for disease management, from combating microbial pathogens to treating diseases by delivering therapeutic molecules and regrowing tissues. The recent issuance of patents with therapeutic applications of synthetic gene circuits indicates the growing interest and investment in research that will help bring these inventions to a next level. While we continue to learn about these technologies and improve them, it is essential to maintain the highest ethical and safety guidelines to maximize the benefits for human beings and society.
